# Sex Differences in the Association Between Masticatory Function and Sarcopenia Components: The Shizuoka Study

**DOI:** 10.3390/nu17060968

**Published:** 2025-03-10

**Authors:** Sayaka Nagao-Sato, Osamu Kushida, Yasunari Kurita, Etsuko Ozaki, Nagato Kuriyama, Michitaka Kato, Rie Akamatsu, Toshinao Goda, Yasuharu Tabara

**Affiliations:** 1 Department of Nutrition, Faculty of Health and Welfare, Takasaki University of Health and Welfare, Gunma 370-0033, Japan; sato-s@takasaki-u.ac.jp; 2 Graduate School of Humanities and Sciences, Ochanomizu University, Tokyo 112-8610, Japan; 3 School of Food and Nutritional Sciences, University of Shizuoka, Shizuoka 422-8526, Japan; kushida@u-shizuoka-ken.ac.jp (O.K.); gouda@u-shizuoka-ken.ac.jp (T.G.); 4 Department of Shizuoka Physical Therapy, Faculty of Health Science, Tokoha University, Shizuoka 420-0831, Japan; ykurita@sz.tokoha-u.ac.jp (Y.K.); katomanzooo@sz.tokoha-u.ac.jp (M.K.); 5 Department of Epidemiology for Community Health and Medicine, Kyoto Prefectural University of Medicine, Kyoto 602-8566, Japan; ozaki@koto.kpu-m.ac.jp; 6 Graduate School of Public Health, Shizuoka Graduate University of Public Health, Shizuoka 420-0881, Japan; nkuriyama@s-sph.ac.jp; 7 Natural Science Division, Faculty of Core Research, Ochanomizu University, Tokyo 112-8610, Japan; akamatsu.rie@ocha.ac.jp; 8 Center for Genomic Medicine, Kyoto University Graduate School of Medicine, Kyoto 606-8501, Japan

**Keywords:** masticatory function, skeletal muscle index, physical performance, body mass index, older adults

## Abstract

**Background/Objectives**: Low masticatory function has been proposed as a risk factor for sarcopenia. This study investigated its potential association with body composition and physical performance in community-dwelling older adults. **Methods**: Participants included adults aged ≥65 years (643 men; 797 women) who volunteered for a longitudinal cohort study. Masticatory function was objectively assessed using gummy jelly and subjectively evaluated via a structured questionnaire. The skeletal muscle mass, body mass index, waist circumference, handgrip strength, gait speed, and five-time chair-stand test were assessed. **Results**: Objectively measured masticatory function was classified as low (12.6%), moderate (38.0%), or high (49.4%). In men, masticatory function was significantly associated with skeletal muscle mass index (low, moderate, and high: 7.4, 7.7, and 7.7 kg/m^2^; *p* = 0.005), handgrip strength (31.8, 34.2, and 35.5 kg; *p* < 0.001), and gait speed (1.3, 1.4, and 1.4 m/s; *p* = 0.003). In women, low masticatory function was linked to a higher body mass index (22.9, 22.0, and 21.9 kg/m^2^; *p* = 0.028) and waist circumference (82.9, 80.8, and 80.4 cm; *p* = 0.041). Moreover, these significant associations persisted after adjusting for covariates. Discrepancies were observed between objective and subjective measures of masticatory function, with approximately 40% of participants showing low objective masticatory function, perceiving their chewing ability as normal. Subjective masticatory function exhibited no significant associations with anthropometric measures or sarcopenia components. **Conclusions**: In men, objectively measured low masticatory function was associated with reduced skeletal muscle mass and poor physical performance; in women, it was linked to higher body mass index.

## 1. Introduction

Oral disorders and diseases cause physical impairments that lead to functional limitations, disabilities, and handicaps [1], which in turn reduce quality of life. Decreased masticatory function is a major phenotype of oral disorders, which is characterized by reduced occlusal force and motor ability of the tongue, leading to chewing difficulties [2]. Although this decline has been well documented as a risk factor for malnutrition [3], it may also increase the risk of systemic sarcopenia in older adults [4,5]. However, previous findings for the association between masticatory function and sarcopenia were inconsistent, i.e., cross-sectional and longitudinal studies have reported associations between subjective and/or objective declines in masticatory function and sarcopenia [6,7,8]. However, when analyzing individual components of sarcopenia, i.e., reduced skeletal muscle mass, weak muscle strength, and impaired physical performance, the results of the association analysis with masticatory function remained inconsistent [9,10,11,12,13,14,15,16,17,18].

One potential explanation for these discrepancies is sex differences in masticatory function and sarcopenia indices. It is well known that skeletal muscle mass, strength, and performance vary substantially between men and women [19]. Chewing function also differs by sex, with women chewing common foods more slowly, using less force, and exhibiting a narrower mouth opening compared with men [20]. Consequently, subjective and objective masticatory function measures are typically better in older men than in older women [15]. Furthermore, sex differences in food preferences, particularly among individuals with chewing difficulties, have been reported [21]. These findings emphasize the importance of sex-separated analyses to better understand the relationship between masticatory function and sarcopenia indices. However, only one study [15] has conducted such analysis, whereas others [9,10,11,13,14,16,17,18] addressed sex differences by including sex as a covariate in statistical models.

Given these backgrounds, we aimed to investigate the associations between masticatory function and sarcopenia components in community-dwelling older adults using sex-separated analyses and objective measures of masticatory function.

## 2. Materials and Methods

### 2.1. Study Participants

This cross-sectional study used data from the Shizuoka study, a longitudinal cohort study of community residents living in Shizuoka Prefecture, Japan. Physically independent Japanese individuals aged 40–85 years enrolled in National Health Insurance (a health insurance system for individuals not eligible for employee-based health insurance) or the Latter-Stage Elderly Medical Care System (a health insurance system for adults aged ≥ 75 years) were eligible for participation. Data for this study were collected during the baseline survey conducted between December 2021 and March 2024 in the Kamo region and Fukuroi City, Shizuoka Prefecture.

Of 1825 participants aged ≥ 65 years, 1440 were included in the final analysis after excluding individuals who met the following criteria: not performing a masticatory function test (*n* = 369), which was performed after December 2022, and lacking data for the nutritional survey (*n* = 4), skeletal muscle mass index (SMI) (*n* = 9), or at least one physical performance measure (*n* = 20). Written informed consent was obtained from all participants. The Ethics Committee of Kyoto University Graduate School of Medicine approved all study procedures for the Shizuoka study (G1327).

### 2.2. Measurement of Objective and Subjective Masticatory Function

Objective masticatory function was assessed using a commercially available gummy jelly (approximately 20 mm wide, 20 mm deep, and 10 mm high; UHA Mikakuto Co., Ltd., Osaka, Japan) [22]. Participants chewed the gummy jelly 30 times and then expelled the comminuted pieces into a plastic dish. The original method assessed masticatory function by measuring glucose dissolved in saliva from the gummy jelly [23] or grading fragmentation using a 10-stage visual scale [23]. In the present study, we counted the number of comminuted pieces by analyzing photographs using ImageJ software, version 1.54 [24].

Subjective masticatory function was evaluated using a self-administrated question, “Which of the following conditions describes your chewing ability during meals?”, with three response options: (1) I can eat anything; (2) it is sometimes difficult to chew due to dental issues, such as dental caries or periodontal disease; or (3) I can hardly chew. This question is included in the questionnaire commonly used in the Specific Health Checkup, which was designed to be performed annually for enrollees in National Health Insurance or the Latter-Stage Elderly Medical Care System.

### 2.3. Assessment of Skeletal Muscle Mass and Physical Performance

SMI was calculated by dividing appendicular lean mass by the square of body height. Appendicular lean mass was estimated using a segmental multifrequency bioelectrical impedance analysis device (MC-780A, TANITA, Tokyo, Japan) at frequencies of 5, 50, and 250 kHz, with values from 50 kHz used for analysis. Handgrip strength was measured using either a Jamar hand dynamometer (MT-230, Sakaimed, Tokyo, Japan) in the Kamo region baseline survey or a Smedley-type hand dynamometer (TKK5401, Takei Scientific Instruments, Niigata, Japan) in the Fukuroi City survey; both devices report grip strength to one decimal place. Measurements were taken twice per hand in a seated position with arms on a desk and horizontal to the ground. The maximum value from all measurements was used for analysis. Gait speed was measured on a 10 m walkway, with the time between the 2 and 8 m marks recorded using two pairs of wireless infrared photocells (Brower Timing Systems, Draper, Utah, USA). Measurements were taken twice, and the average was used for analysis. The five-time chair-stand test was conducted using a 43 cm high chair without armrests. Participants were instructed to stand up five times as quickly as possible, starting from a seated position with arms crossed over their chest. The time from the initial sitting position to the fifth stand was recorded. This test was performed once.

### 2.4. Dietary Intake Assessment

Dietary intake was evaluated using a structured self-administered diet history questionnaire [25], which assessed the frequency of consuming 50 foods and beverages over the past month. Using the consumption data, an ad hoc algorithm estimated intakes of macronutrients and micronutrients. Among them, dietary intakes of energy, protein, fat, and carbohydrates were used in the current study. The validity and reproducibility of this questionnaire have been reported previously [26].

### 2.5. Basic Clinical Parameters

Other basic clinical parameters were obtained from the baseline survey of the Shizuoka study. Waist circumference was measured at the umbilical level.

### 2.6. Statistical Analysis

Data are presented as means (with standard deviations) or frequencies. Sex differences in objective masticatory function, represented by the number of fragmented gummy jelly pieces and three classification groups, were tested using the *t*-test and Fisher’s exact test, respectively. Associations between objective and subjective masticatory function were analyzed using Fisher’s exact test. Differences in numeric variables across masticatory function groups were assessed using analysis of variance. Multiple linear regression models were used to determine whether masticatory function independently predicted physical performance measures. Moreover, each regression model included a physical performance variable as the dependent variable and masticatory function as the independent variable, with adjustments for age, related physical performance variables, and dietary intake. Statistical significance was set at *p* < 0.05. Analyses were performed using JMP Pro, version 17.0.0 (SAS Institute Inc., Cary, NC, USA).

## 3. Results

**Figure 1:**
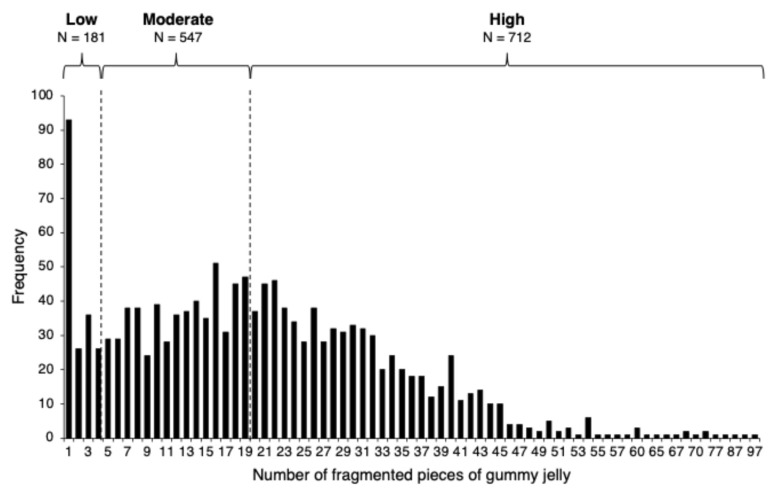
Distribution of fragmented gummy jelly pieces.

Table 1 presents the clinical characteristics of the study participants, including 643 (44.7%) men and 797 (55.3%) women. The distribution of comminuted pieces is illustrated in Figure 1. According to a 10-stage visual rating scale [27], masticatory function was categorized as low (*n* = 181, 12.6%) if four pieces or fewer were fragmented, whereas the median number of pieces (nineteen) was used as the cutoff between moderate (*n* = 547, 38.0%) and high (*n* = 712, 49.4%) masticatory function. Owing to sex differences in the mean number of comminuted pieces (*t* = −5.86, *p* < 0.001; Table 1), significant differences were found between men and women in the frequency of high (men: 25.3%; women: 24.1%), moderate (men: 14.9%; women: 23.1%), and low (men: 4.4%; women: 8.2%) masticatory function (*χ*^2^ = 26.0, *p* < 0.001).

**Figure 2:**
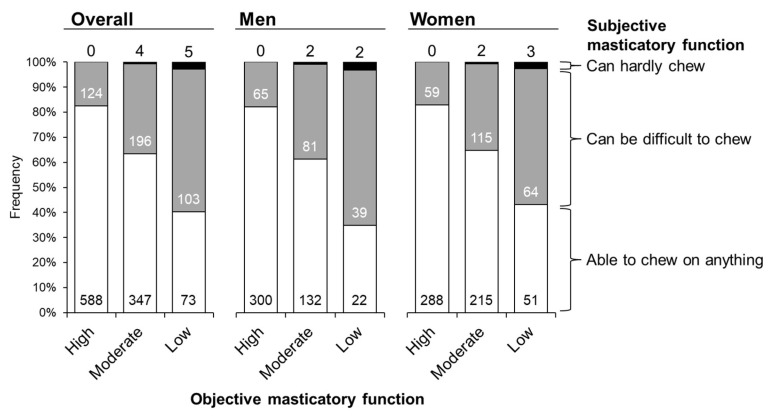
Associations between objective and subjective masticatory function.

Figure 2 shows the associations between subjective masticatory function, assessed via a questionnaire, and objective masticatory function, measured using the gummy jelly. Despite having a low objective masticatory function, approximately 40% of participants perceived themselves as able to chew anything overall.

Objective function was evaluated using gummy jelly, whereas subjective function was assessed using a structured questionnaire. Furthermore, frequency differences were evaluated using Fisher’s exact test (all *p* < 0.001).

Differences in anthropometric and physical performance measures across the masticatory function groups are summarized in Table 2. Results of the multiple linear regression analysis for each component of sarcopenia are presented in Table 3. The regression analysis indicated that low objective masticatory function was independently associated with low SMI in men, even after adjusting for total protein intake. Low objective masticatory function was also associated with weak handgrip strength and slow gait speed in men, independent of body mass index (BMI) and SMI. Conversely, low objective masticatory function in women was associated with high BMI and waist circumference but not with any components of sarcopenia. No significant association was found between subjective masticatory function and anthropometric factors or any component of sarcopenia.

## 4. Discussion

This study examined the associations between objectively measured masticatory function and the components of sarcopenia in older men and women. Low masticatory function was associated with lower skeletal muscle mass, weaker handgrip strength, and slower gait speed in men. In women, low masticatory function was associated with higher BMI and larger waist circumference but not with sarcopenia components. Subjective masticatory function was not always consistent with the objective measurements and showed no association with anthropometric factors or sarcopenia components.

In previous studies, five studies have assessed objective masticatory function [13,14,15,16,18]. As for handgrip strength, two studies [13,14] found that greater strength was associated with better objective masticatory function, while another two studies [15,16] reported a lack of association. This discrepancy may partly be derived from the proportion of men in the study population. The proportion of men in the former studies was 50.3% and 44.1% [13,14], while that in the latter studies [15,16] was 37.1% and 42.9%. A similar tendency was observed in the studies of gait speed; namely, a study reported a lack of association based on the analysis of a population comprising 42.9% men [16]. No clear differences in the proportion of men were observed between studies that reported a significant association between masticatory function and skeletal muscle mass and studies that reported a lack of association [14,16,18]. However, given the lower proportion of men in the studies reporting significant associations with physical performance measures [13,14], sex-separated analysis is warranted in studies aiming to clarify the association between masticatory function and sarcopenia components.

Several studies have reported sex differences in age-related changes in muscle mass and physical performance measures [28,29,30,31,32], although the results were inconsistent. Typically, men experience a greater age-related decline in physical performance [28,30,31] and skeletal muscle mass [29,32] compared with women. The reason why masticatory function was associated with skeletal muscle mass and physical performance measures only in men remains unclear, but it may be partly due to the more pronounced decline in muscle mass and function in men. A possible mediator linking low masticatory function and reduced muscle mass is decreased protein intake. Individuals with reduced masticatory function tend to consume fewer crunchy foods, such as fruits and vegetables, and more processed foods with lower protein content [33]. However, the association between low masticatory function and skeletal muscle mass remained significant even after adjusting for protein intake. As protein intake was estimated using a structured questionnaire, more precise dietary measurements could help clarify the nutritional factors involved in this relationship. Alternatively, the loss of masseter muscle mass, which may occur alongside systemic muscle mass loss, could contribute to the link between masticatory function and skeletal muscle mass. Low masticatory function was also associated with poor physical performance. These associations, independent of skeletal muscle mass, suggest that muscle quality, particularly the fat infiltration into the muscle, may play a role in the relationship.

A positive correlation was observed between subjective and objective masticatory functions, consistent with the findings of a previous study of Japanese older adults, where participants with better subjective masticatory function were more likely to have better objective function [16]. However, a large gap existed between subjective and objective assessments, with approximately 40% of participants who exhibited low objective masticatory function reporting that they were “able to chew anything”. This gap may be due to a preference for easy-to-chew foods among individuals with low masticatory function [33]. Additionally, subjective masticatory function was not associated with anthropometric factors or physical performance measures. These findings underscore the importance of using objective measurements in the assessment of masticatory function in epidemiological studies and community health practice.

This study has limitations. First, causality between masticatory function and anthropometric factors and sarcopenia components cannot be established owing to the cross-sectional nature of the study. Second, as the study population consisted of physically independent community residents who volunteered to participate, the masticatory function of this group may differ from that of the general population due to healthy participant bias.

## 5. Conclusions

Objectively measured low masticatory function, but not subjective assessment, was interpedently associated with low skeletal muscle mass, weak handgrip strength, and slow gait speed in men and high BMI in women. Objective measurements are essential for accurately assessing masticatory function.

## Figures and Tables

**Table 1:** Clinical characteristics of the study participants (*n* = 1440).

	Overall	Men	Women
*n*	1440	643	797
Age, years	73.9 (4.6)	74.1 (4.5)	73.8 (4.6)
Body mass index, kg/m^2^	22.6 (3.2)	23.2 (2.9)	22.1 (3.4)
Waist circumference, cm	82.7 (9.3)	84.9 (8.7)	80.9 (9.4)
Sarcopenia components			
Skeletal muscle mass index, kg/m^2^	7.0 (0.9)	7.7 (0.7)	6.4 (0.5)
Handgrip strength, kg	27.9 (8.3)	34.7 (6.6)	22.4 (4.4)
Gait speed, m/s	1.4 (0.2)	1.4 (0.2)	1.4 (0.2)
Chair-stand test, s	7.4 (2.1)	7.4 (2.2)	7.4 (2.0)
Dietary intake			
Total energy, kcal	1843 (523)	2019 (543)	1701 (461)
Protein, g	77 (28)	80 (29)	74 (27)
Fat, g	56 (20)	59 (21)	54 (19)
Carbohydrate, g	240 (70)	261 (72)	224 (63)
Masticatory function			
Fragmented pieces of gummy jelly, *n*	20.6 (13.8)	23.0 (14.4)	18.7 (12.9)
Self-assessment, *n*			
Able to chew on anything	1008	454	554
Can be difficult to chew	423	185	238
Can hardly chew	9	4	5

Values are means (with standard deviations) or frequencies.

**Table 2:** Differences in sarcopenia characteristics according to masticatory function.

	Masticatory Function		*n*	Age, Years Old	Body Mass Index, kg/m^2^	Waist Circumference, cm	Skeletal Muscle Mass Index, kg/m^2^	Handgrip Strength, kg	Gait Speed, m/s	Five-Time Chair-Stand Test Time, s
Men	Objective	Low	63	75.4 (0.6)	23.0 (0.4)	84.4 (1.1)	7.4 (0.09)	31.8 (0.8)	1.3 (0.03)	7.7 (0.3)
		Moderate	215	74.3 (0.3)	23.4 (0.2)	84.9 (0.6)	7.7 (0.05)	34.2 (0.4)	1.4 (0.02)	7.5 (0.2)
		High	365	73.7 (0.2)	23.2 (0.2)	84.9 (0.5)	7.7 (0.04)	35.5 (0.3)	1.4 (0.01)	7.3 (0.1)
		*p*		0.014	0.630	0.904	0.005	<0.001	0.003	0.337
	Subjective	Can hardly chew	4	68.8 (2.2)	24.8 (1.5)	89.0 (4.3)	7.7 (0.37)	32.2 (3.3)	1.2 (0.11)	7.5 (1.1)
		Can be difficult to chew	185	74.1 (0.3)	23.4 (0.2)	85.2 (0.6)	7.7 (0.05)	34.9 (0.5)	1.4 (0.02)	7.6 (0.2)
		Able to chew on anything	454	74.1 (0.2)	23.2 (0.1)	84.7 (0.4)	7.7 (0.03)	34.7 (0.3)	1.4 (0.01)	7.4 (0.1)
		*p*		0.061	0.466	0.536	0.974	0.714	0.021	0.701
Women	Objective	Low	118	75.0 (0.4)	22.9 (0.3)	82.9 (0.9)	6.4 (0.04)	21.9 (0.4)	1.4 (0.02)	7.9 (0.2)
		Moderate	332	74.1 (0.3)	22.0 (0.2)	80.8 (0.5)	6.3 (0.03)	21.8 (0.2)	1.4 (0.01)	7.6 (0.1)
		High	347	73.1 (0.2)	21.9 (0.2)	80.4 (0.5)	6.4 (0.03)	23.0 (0.2)	1.5 (0.01)	7.1 (0.1)
		*p*		<0.001	0.028	0.041	0.129	0.001	0.004	<0.001
	Subjective	Can hardly chew	5	74.6 (2.1)	23.1 (1.5)	85.4 (4.2)	6.5 (0.22)	21.4 (2.0)	1.5 (0.10)	6.8 (0.9)
		Can be difficult to chew	238	74.2 (0.3)	22.0 (0.2)	80.6 (0.6)	6.3 (0.03)	22.0 (0.3)	1.4 (0.01)	7.6 (0.1)
		Able to chew on anything	554	73.6 (0.2)	22.1 (0.1)	81.0 (0.4)	6.4 (0.02)	22.5 (0.2)	1.5 (0.01)	7.4 (0.1)
		*p*		0.197	0.757	0.494	0.312	0.204	0.056	0.137

Values are means (with standard deviations). Statistical significance was determined using analysis of variance.

**Table 3:** Multiple linear regression analysis of anthropometric and sarcopenic factors.

Masticatory Function		Body Mass Index ^1^		Waist Circumference ^1^		Skeletal Muscle Mass Index ^2^		Handgrip Strength ^3^		Gait Speed ^3^		Five-Time Chair-Stand Test Time ^3^	
		*β*	*p*	*β*	*p*	*β*	*p*	*β*	*p*	*β*	*p*	*β*	*p*
Men	Objective												
	Low	−0.151	0.561	−0.274	0.724	−0.126	**0.013**	−0.982	**0.049**	−0.047	**0.020**	0.054	0.783
	Moderate	0.170	0.366	0.160	0.775	0.047	0.196	0.058	0.871	0.011	0.458	0.023	0.873
	High	reference		reference		reference		reference		reference		reference	
	Subjective												
	Can hardly chew	0.824	0.401	2.455	0.401	−0.223	0.243	−2.879	0.126	−0.141	0.061	0.188	0.800
	Can be difficult to chew	−0.327	0.522	−1.012	0.506	0.099	0.321	1.536	0.117	0.053	0.175	−0.017	0.965
	Able to chew on anything	reference		reference		reference		reference		reference		reference	
Women	Objective												
	Low	0.610	**0.007**	1.584	**0.012**	−0.029	0.308	−0.085	0.747	−0.018	0.192	0.239	0.060
	Moderate	−0.253	0.140	−0.618	0.199	−0.012	0.567	−0.306	0.125	−0.002	0.812	0.063	0.513
	High	reference		reference		reference		reference		reference		reference	
	Subjective												
	Can hardly chew	0.795	0.432	3.231	0.253	0.032	0.796	−0.694	0.554	0.055	0.369	−0.522	0.358
	Can be difficult to chew	−0.426	0.418	−1.789	0.224	−0.033	0.618	0.209	0.732	−0.043	0.179	0.368	0.213
	Able to chew on anything	reference		reference		reference		reference		reference		reference	

^1^ Adjusted for age and total energy intake; ^2^ adjusted for age, body mass index, and protein intake; ^3^ adjusted for age, body mass index, and skeletal muscle mass index. Bold text indicates significant *p* values at < 0.05.

## Data Availability

Data are available on reasonable request due to ethical reasons.

## Abbreviations

The following abbreviations are used in this manuscript:

SMI	skeletal muscle mass index
BMI	body mass index
